# Do frail elderly people affect the nursing workload in intensive care units?^*^

**DOI:** 10.1590/1980-220X-REEUSP-2021-0599en

**Published:** 2022-11-14

**Authors:** Fabiola Mika Tanabe, Suely Sueko Viski Zanei, Iveth Yamaguchi Whitaker

**Affiliations:** 1 Universidade Federal de São Paulo, Escola Paulista de Enfermagem, São Paulo, SP, Brazil.

**Keywords:** Frail Elderly, Nursing, Workload, Critical Care, Intensive Care Units, Anciano Frágil, Enfermería, Carga de Trabajo, Cuidados Críticos, Unidades de Cuidados Intensivos, Idoso Fragilizado, Enfermagem, Carga de Trabalho, Cuidados Críticos, Unidades de Terapia Intensiva

## Abstract

**Objective:**

Compare the nursing workload according to the condition of frailty in elderly people in an intensive care unit (ICU).

**Method:**

A cross-sectional study whose sample included patients aged ≥60 years who were hospitalized for ≥24 hours in the ICU of a university hospital in São Paulo, Brazil. The Tilburg Frailty Indicator (TFI) was used to identify frailty in elderly people and the Nursing Activities Score (NAS) was used to measure the nursing workload.

**Results:**

In a sample of 204 elderly people, frailty was found in 156 (76.5%). The elderly people contributed to high nursing workload (mean NAS 75.9) on the first day in the ICU, but frailty did not significantly change the NAS (p = 0.606) (frail 75.7 versus non-frail 76.5), either based on the mean value or the proportion of patients in each category.

**Conclusion:**

The condition of frailty in elderly people did not increase the nursing workload in the ICU.

## RESUMO

**Objetivo:**

Comparar a carga de trabalho de enfermagem de idosos de acordo com a condição de fragilidade em unidade de terapia intensiva (UTI).

**Método:**

Estudo transversal cuja amostra incluiu pacientes com idade ≥60 anos e internação ≥24h na UTI de hospital universitário de São Paulo, Brasil. *Tilburg Frailty Indicator* (TFI) foi aplicado para identificar fragilidade no idoso e o *Nursing Activities Score* (NAS) para mensurar carga de trabalho de enfermagem.

**Resultados:**

Na amostra com 204 idosos, a condição de fragilidade foi constatada em 156 (76,5%). Os idosos demandaram alta carga de trabalho de enfermagem (NAS médio 75,9) no primeiro dia na UTI, mas a fragilidade não alterou significativamente (p = 0,606) o NAS (frágil 75,7 vs não frágil 76,5), seja considerando a média seja a proporção de pacientes em cada categoria.

**Conclusão:**

A condição de fragilidade em idosos não repercutiu no aumento da carga de trabalho de enfermagem em UTI.

## RESUMEN

**Objetivo:**

Comparar la carga de trabajo de enfermería de ancianos según su condición de fragilidad en unidad cuidado intensivo (UCI).

**Método:**

Estudio transversal con muestra incluyendo pacientes con edad ≥60 años e internación ≥24 hs en UCI de hospital universitario de São Paulo, Brasil. Se aplicó el *Tilburg Frailty Indicator* (TFI) para identificar fragilidad en los ancianos y el *Nursing Activities Score* (NAS) para medir carga de trabajo de enfermería.

**Resultados:**

En la muestra de 204 ancianos, la condición de fragilidad fue constatada en 156 (76,5%). Los ancianos demandaron alta carga de trabajo de enfermería (NAS promedio: 75,9) en el primer día en UCI, pero la fragilidad no cambió significativamente (p = 0,606) el NAS (frágil 75,7 vs. no frágil 76,5), tanto considerando la media o la proporción de pacientes en cada categoría.

**Conclusión:**

La condición de fragilidad en ancianos no repercutió en el aumento de carga de trabajo de enfermería en UCI.

## INTRODUCTION

With the aging of the population, hospitalization of elderly people with changes related to the senescence and/or senility processes is frequently observed^(1)^. The frailty syndrome has attracted the attention of researchers because it has significantly increased among the elderly and is characterized by biological and psychosocial changes, especially in physical aspects, which can cause disproportionate impairment due to the poor physiological response to stressors, leading patients to a condition of extreme vulnerability^(2)^. From a physiological perspective, frailty refers to the process of functional reserve decline in an individual, causing loss of the ability to maintain homeostasis, resulting in difficult recovery from diseases^(3)^.

Given the high prevalence of hospitalized elderly people, also in intensive care units (ICUs), the possibility of frailty and severe clinical conditions in the elderly increases^(4)^. Older people with frailty and illnesses severity in the ICU with clinical therapies and complex care may require more time from the nursing team and increase their workload. Therefore, an increase in hospitalization of elderly people in the ICU, especially frail elderly, can impact the workload and affect the dynamics of the nursing team and the quality of care.

The complexity of technologies for monitoring and treatment of critically ill patients can directly affect nursing care and, consequently, increase the nursing workload. Nursing workload is defined as the “amount of time and care directly or indirectly dedicated to patients, workplace and professional development.” This definition is based on five attributes: “nursing time; level of knowledge, skills, and behavior; nursing intensity weight; physical, mental, and emotional process, and the ability to change the plan during the shift”^(5)^.

One of the tool that measure the time spent with nursing care in the ICU, which reflects the workload, is the Nursing Activities Score (NAS)^(6,7)^. The workload of every patient is expressed as a percentage, indicating the amount of time in the work shift spent to perform the required care^(6,7)^.

The analysis of the nursing workload required for elderly care in the ICU, which was measured with the NAS in three hospitals in the city of São Paulo, showed an overall mean of 72.9%, and patients aged 70 to 79 years with higher illness severity were more likely to demand a high workload than patients who were 10 years younger^(8)^. In another study, conducted at a university hospital in Londrina, Paraná, no significant difference was observed in the NAS presented by elderly patients (73.99 to 74.3%) and non-elderly patients (74.08 to 75.18%)^(9)^, that is, the scores indicated high nursing workload in both groups. The analysis of the nursing workload in an ICU in Portugal using the Simplified Therapeutic Intervention Scoring System (TISS-28) also showed that aging was directly proportional to workload increase, with higher scores observed in the elderly when compared to other age groups of adulthood (p < 0.001)^(10)^. Although the workload was measured with another tool in our study, it is important to emphasize the TISS-28 was the precursor of the NAS^(7)^, whose measurement is based on items related to care complexity, comprised of categories of therapeutic intervention that remained in the NAS.

Although the studies mentioned above indicated a high nursing workload when providing care to elderly people in the ICU, it is important to emphasize that the frailty condition of the elderly was not considered in these analyses. The possibility of physical impairment in frailty to increase the severity of the clinical condition of the elderly, requiring complex therapy and long-term nursing care, raised the following question: is the frailty condition of the elderly associated with the nursing workload in the ICU?

Frailty is a common multidimensional condition among the elderly associated with adverse responses to diseases^(11,12)^. Inadequate human resource allocation in nursing care directly affects the quality of care provided, negatively impacting quality indicators such as infection, falls, medication errors, costs, and length of stay^(13)^, as nursing activities are influenced by the degree of disease complexity and patient characteristics. Therefore, the importance of this study is explained by the need to understand the impact of frail elderly patients on the nursing workload to allow individualized care with safety and quality.

Considering the prevalence of elderly patients in ICUs and the lack of studies assessing the impact of frailty on the time of care provided by the nursing team to elderly patients, this study aimed to analyze the nursing workload related to the condition of frailty in elderly patients in the ICU.

## METHOD

### Study Design and Setting

This observational cross-sectional study was conducted in the two general ICUs (26 beds) and one neurology ICU (9 beds), of Hospital São Paulo (HSP), a large hospital linked to the Federal University of São Paulo (Unifesp) as a university hospital, located in the city of São Paulo, Brazil.

### Sampling and Selection Criteria

The study sample was selected by convenience sampling method and consisted of patients who met the following inclusion criteria: age ≥60 years, who had been in the ICU for more than 24 hours. Patients receiving palliative care as stated by the medical team were not included in the study, since the clinical therapy and patient monitoring approaches defined in this condition could affect the nursing workload, leading to reduced NAS and becoming a confounding factor. Patients were not reintroduced in the study in case of readmission after the start of data collection. Sedated or comatose patients whose caregivers or legal representatives did not attend the ICU visiting hours were considered losses and not included in the study, as the informed consent form could not be signed.

### Data Collection

Data collection was conducted from October 2017 to April 2018 by three residents of the multiprofessional residency program in adult intensive care unit. Residents had data collection training where they received information about the Tilburg Frailty Indicator (TFI)^(14)^, the NAS manual, and the data collection form. Considering the NAS is often used in ICUs, the NAS manual was revised. After this stage, the practical training was focused on the application of the TFI and the NAS by the residents to patients selected by two professors of the program. The scores obtained were compared and discussed until there was no divergence and doubt regarding the application of these instruments.

During the data collection period, 585 patients were admitted to the ICU; of those, 333 (56.8%) were elderly and 204 (61.3%) were included in the study. Of all 129 (38.7%) patients that were not selected, 54 (41.9%) were readmitted, 29 (22.5%) stayed in the ICU for ≤ 24 hours, 28 (21.7%) patients and/or family members refused to participate in the study, 15 (11.6%) had no informed consent form, and 3 (2.3%) were in palliative care. Sociodemographic and clinical data of the patients were collected from their medical records and added to a specific form.

### Variables

The following variables were studied: age, sex (male and female), ICU and hospital discharge status, admission category, length of stay, comorbidities (Charlson Comorbidity Index)^(15)^, illness severity (Simplified Acute Physiology Score III – SAPS 3)^(16)^, frailty condition (TFI)^(14)^, and nursing workload (NAS)^(6,7)^.

The TFI consists of two sections (A and B). Section A analyzes the determinants of frailty and section B aims to identify frailty itself. It has 15 self-reported and objective questions about the physical, psychological, and social domains of frailty. The score can range from 0 to 15, in which scores of 5 or more indicated the presence of frailty^(14)^. It was selected for this study because it is a multidimensional tool, addressing the physical, psychological, and social dimensions of frailty. The conditions of the elderly considered in the TFI referred to the period of six months before their admission to the ICU.

The TFI was applied to conscious and oriented patients, who were clearly explaining that the studied conditions were related to the period of six months before hospital admission. For sedated or comatose patients, the TFI was answered by a proxy, who was a family member or caregiver spending at least 8 hours a day with the elderly for at least three months^(17)^. The TFI was answered by 105 (51.5%) patients and 99 (48.5%) proxies.

The NAS, an instrument used to measure the nursing workload in the ICU, has seven categories and 23 items (1: Monitoring and titration, 2: Laboratory tests, 3: Medication, 4: Hygiene procedures, 5: Care of drains, 6: Mobilization and positioning, 7: Support and care of relatives and patient, 8: Administrative and managerial tasks, 9–11: Ventilatory support, 12–15: Cardiovascular support, 16–17: Renal support, 18: Neurological support, 19–21: Metabolic support, 22-23: Specific interventions) corresponding to the direct and indirect care needs of critically ill patients^(6)^. The sum of the points assigned to each category results in a total score, expressed as a percentage, which represents the time spent with nursing care in the ICU for each patient in the last 24 hours. If the total score is 100, the patient required 100% of the time of nursing staff in the last 24 hours. Each NAS point is equivalent to 14.4 minutes of nursing care. The NAS was applied to every patient once a day in their first week in the adult intensive care unit (AICU)^(18)^. NAS application in the first week of the ICU was defined considering the average length of stay in Brazilian ICUs^(19)^. NAS scores above 71.1% were considered high nursing workload^(8)^. For the nursing workload analysis, the highest NAS mean was selected, considering that it can represent a moment of higher instability and severity of the clinical condition of the elderly, requiring immediate treatment and nursing care, leading to direct or indirect activities^(20)^.

### Data Analysis and Treatment

In the descriptive analysis, qualitative variables were processed as absolute and relative numbers and quantitative variables as mean and standard deviation values. The Mann-Whitney test was used to compare the mean NAS scores of the first seven days and the mean SAPS 3 scores between frail and non-frail elderly patients, using the Shapiro-Wilk test to check for abnormal distribution. The chi-square or Fisher’s exact test was applied to compare the proportion of elderly people in the groups according to the frailty condition, in each NAS category/item, and the assigned score. The significance level adopted in the statistical analyses was 95% (p ≤ 0.05).

### Ethical Aspects

The study project was approved in 2017 by the Research Ethics Committee of the Federal University of São Paulo (CEP/Unifesp), document CEP/Unifesp 2.330.740, per Resolution no. 510/2016 of the National Health Council. Informed consent forms were signed by the patients or their legal representatives whenever they were unable to sign them.

## RESULTS

The sample consisted of 204 elderly people, and frailty was found in 156 (76.5%) of them. The mean age of the elderly patients was 72.5 years (min.: 60; max.: 97; median: 71.0; SD: 8.3); 50.5% of them were male, most in the surgical admission category (61.6%), with a mean Charlson Comorbidity Index of 2.2 (min.: 0; max.: 11; median: 2.0). They stayed in the ICU for an average of 9.2 days (min.: 1; max.: 76; median: 5; SD: 11.73), and mortality was 19.1%. The overall mean in SAPS 3 was 50.9 (min.: 23; max.: 94; median: 49; SD: 13.41), and the mean of frail elderly was higher (52.1; min.: 25; max.: 94; median: 50.5; SD: 13.54) than the mean of non-frail elderly (47.1; min.: 23; max.: 76; median: 45.5; SD: 12.35), (p = 0.029).

When comparing the mean values obtained in the NAS (Table 1) according to the frailty condition of the elderly, from day 1 to day 7, no statistical difference was observed between the mean values of frail and non-frail groups. Also, no statistical difference was observed when comparing the overall mean NAS between the two groups of elderly people, considering the condition of frailty.

**Table 1.** Mean and standard deviation of the NAS obtained from the elderly patients in the first week in the ICU, according to the frailty condition. HSP/Unifesp. São Paulo, Brazil 2018.

	Condition				
	Frailty		Non-frailty		
	Mean	Standard deviation	Mean	Standard deviation	p value
**NAS – day 1**	75.7	8.2	76.5	8.5	0.606
**NAS – day 2**	56.9	11.0	56.9	9.0	0.694
**NAS – day 3**	55.0	7.7	57.0	7.7	0.235
**NAS – day 4**	56.8	10.4	58.2	15.6	0.874
**NAS – day 5**	57.0	10.2	58.4	12.1	0.698
**NAS – day 6**	57.4	9.8	54.2	7.4	0.318
**NAS – day 7**	56.9	9.6	56.5	10.7	0.698
**Overall mean NAS**	59.5	7.3	58.8	7.6	0.179

Mann-Whitney test.

The workload on the first day in the ICU reached its highest mean in the NAS; then, the scores of each category/item for that day were selected for the comparison of frail and non-frail groups (Table 2, 3, and 4).

**Table 2.** Monitoring and titration, laboratory tests, medication, hygiene procedures, care of drains, and mobilization and positioning required by the elderly patients according to NAS categories/items and frailty condition. HSP/Unifesp – São Paulo, Brazil, 2018.

NAS categories/items (score)	Condition				p value
	Frail n	%	Non-frail n	%	
**Monitoring and titration**					
1a. Hourly vital signs, regular registration and calculation of fluid balance. (4.5)	–	–	–	–	
1b. Presence at bedside and continuous observation or active for 2 hours or more in any shift, for reasons of safety, severity or therapy, such as: non-invasive mechanical ventilation, weaning procedures, restlessness, mental disorientation, prone position, donation procedures, preparation and administration of fluids and/or medication, assisting specific procedures. (12.1)	156	100	48	100	1
1c. Present at bedside and active for 4 hours or more in any shift for reasons of safety, severity or therapy, such as those examples above. (19.6)					
**Laboratory tests**					
Absent. (0.0)	–	–	–	–	
Present. (4.3)	156	100	48	100	1
**Medication, vasoactive drugs excluded**					
Absent. (0.0)	–	–	–	–	
Present. (5.6)	156	100	48	100	1
**Hygiene procedures**					
4a. Performing hygiene procedures such as: dressing of wounds and intravascular catheters, changing linen, washing patient body in special situations (incontinence, vomiting, burns, leaking wounds, complex surgical dressing with irrigation), special procedures (e.g. barrier nursing, etc. (4.1)	156	100.0	47	97.9	1
4b. The performance of hygiene procedures took more than 2 hours in any shift. (16.5)	–	–	1	2.1	
4c. The performance of hygiene procedures took more than 4 hours in any shift. (20.0)	–	–	–	–	
**Care of drains – All (except gastric tube)**					
Absence. (0.0)	97	62.2	25	52,1	
All, except gastric tube. (1.8)	59	37.8	23	47.9	0.212
**Mobilization and positioning**, including procedures such as: turning the patient; mobilization of the patient; moving from bed to chair; team lifting (e.g. immobile patient, traction, prone position).					
6a. Performing procedure(s) up to 3 times per 24 hours. (5.5)	17	10.9	1	2.1	
6b. Performing procedures(s) more frequently than 3 times per 24 hours, or with 2 nurses – any frequency. (12.4)	139	89.1	47	97.9	0.079*
6c. Performing procedure with 3 or more nurses – any frequency. (17.0)	–	–	–	–	

Chi-square; *Fisher’s exact test.

**Table 3.** Special care required by elderly patients according to NAS administrative categories and frailty condition. HSP/Unifesp – São Paulo, Brazil, 2018.

NAS categories/items (score)	Condition				p value
	Frail n	%	Non-frail n	%	
**Support and care of relatives and patient**, including procedures such as telephone calls, interviews, counseling. Often, the support and care of either relatives or patients allow staff to continue with other nursing activities (e.g.: communication with patients during hygiene procedures, communication with relatives while present at bedside and observing the patient).					
7a. Support and care of either relatives or patients requiring full dedication for about one hour in any shift such as to explain clinical condition, dealing with pain and distress, difficult family circumstances. (4.0)	153	98.1	47	97.9	1*
7b. Support and care of either relatives or patients requiring full dedication for 3 hours or more in any shift such as to inform about death, demanding circumstances (e.g.: large number of relatives, language problems, hostile relatives). (32.0)	3	1.9	1	2.1	
**Administrative and managerial tasks**					
8a. Performing routine tasks such as processing of clinical data, ordering examinations, professional exchange of information (e.g.: ward rounds, clinical visits). (4.2)	–	–	–	–	
8b. Performing administrative and managerial tasks requiring full dedication for about 2 hours in any shift such as research activities, protocols in use, admission and discharge procedures. (23.2).	155	99.4	48	100.0	1		
8c. Performing administrative and managerial tasks requiring full dedication for about 4 hours or more of the time in any shift such as death and organ donation procedures, coordination with other disciplines. (30.0)	1	0.6	–	–			

Chi-square; *Fisher’s exact test.

**Table 4.** Care required by elderly patients according to categories of therapeutic support and interventions of the NAS and frailty condition. HSP/Unifesp. – São Paulo, Brazil, 2018.

NAS categories/items (score)	Condition				p value
	Frail n	%	Non-frail n	%	
**Ventilatory support**					
9. Respiratory support: Any form of mechanical/assisted ventilation with or without positive end-expiratory pressure, with or without muscle relaxants; spontaneous breathing with positive end-expiratory pressure (e.g. CPAP or BiPAP), with or without endotracheal tube; supplementary oxygen by any method. (1.4)					
10. Care of artificial airways: Endotracheal tube or tracheostomy cannula. (1.8)					
11. Treatment for improving lung function: Thorax physiotherapy, incentive spirometry, inhalation therapy, intratracheal suctioning. (4.4)					
Absence (0.0)	73	46.8	21	43.8	
9 and 10	4	2.6	–	–	
9 and 11	5	3.2	1	2.1	
9, 10, and 11	37	23.7	13	27.1	0.635
9	36	23.1	13	27.1	0.569
11	1	0.6	–	–	
**Cardiovascular support**					
12. Vasoactive medication, disregard type and dose. (1.2)					
13. Intravenous replacement of large fluid losses. Fluid administration >3 l/m^2^/day, irrespective of type of fluid administered. (2.5)					
14. Left atrium monitoring: Pulmonary artery catheter with or without cardiac output measurement. (1.7)					
15. Cardiopulmonary resuscitation after arrest, in the past period of 24 hours (single precordial thump not included). (7.1)					
Absence (0.0)	107	68.6	31	64.6	
12 and 14 (1.2 and 1.7)	3	1.9	1	2.1	
12 (1.2)	46	29.5	16	33.3	0.612
**Renal support**					
16. Hemofiltration techniques, dialysis techniques. (7.7)					
17. Quantitative urine output measurement (e.g.: by indwelling urinary catheter). (7.0)					
Absence (0.0)	29	18.6	9	18.8	
16 (7.7)	1	0.6	–	–	
17 (7.0)	123	78.8	39	81.2	0.718
16 and 17 (7.7 and 7.0)	3	1.9	–	–	
**Neurological support**					
Absence (0.0)	146	93.6	46	95.8	0.736*
18. Measurement of intracranial pressure. (1.6)	9	5.8	2	4.2	
**Metabolic support**					
Absence (0.0)	140	89.7	43	89.6	
19. Treatment of complicated metabolic acidosis/alkalosis. (1.3)	15	9.6	5	10.4	1
20. Intravenous hyperalimentation. (2.8)	1	0.6	–	–	1
21. Enteral feeding through gastric tube or other gastrointestinal route (e.g., jejunostomy).	–	–	–	–	
**Specific interventions**					
Absence (0.0)	125	80.1	40	83.3	
22. Specific interventions in the intensive care unit: Endotracheal intubation, insertion of pacemaker, cardioversion, endoscopy, emergency surgery in the previous 24 hours, gastric lavage. Routine interventions without direct consequences to the clinical condition of the patient, such as: radiographs, echography, electrocardiogram, dressings, or insertion of venous or arterial catheters are not included. (2.8)	21	13.5	3	6.2	0.175
23. Specific interventions outside the intensive care unit: Surgery or diagnostic procedures. (1.9)	7	4.5	5	10.4	0.158*
22 and 23 (1.9 and 2.8)	3	1.9	–	–	

Chi-square; *Fisher’s exact test.

Table 2 shows that in the categories/items “monitoring and titration,” “laboratory tests,” “medication,” “hygiene procedures,” “care of drains,” and “mobilization and positioning,” the proportion was the same for the total number of frail and non-frail elderly patients.

Regarding the category “support and care of relatives and patient” (Table 3), sub-item 7b, which addresses support and care of either relatives or patient requiring full dedication for 3 hrs or more in any shift, was observed for a higher proportion of frail elderly people, but without statistically significant difference. Performing administrative and managerial tasks that require full dedication for about 4 hours or more in any shift was observed for only one frail elderly person.

Table 4 shows the distribution of elderly people according to the different therapeutic supports and interventions considered in the NAS.

The frequency of elderly people who needed care according to the therapeutic support considered in the NAS showed that elderly patients with frailty did not require a higher nursing workload. However, in some types of support and interventions, a higher frequency of frail elderly was observed, as explained below.

Frail elderly people received scores in the “ventilatory support” category for using any form of mechanical/assisted ventilation, as they required artificial airways and treatment to improve lung function, such as endotracheal aspiration. These sub-items were recorded both mutually (p = 0.635) and individually (p = 0.569), without showing statistical differences between the groups of elderly people.

In the “renal support” category, both frail and non-frail elderly patients required quantitative urine output measurements (17), such as the use of an indwelling urinary catheter. However, when evaluating item 16 (hemofiltration techniques) alone and items 16 and 17 together, only frail elderly patients had these records.

For intravenous hyperalimentation (20), only one frail elderly patient had such records.

Regarding specific interventions, frail elderly patients were submitted to more specific interventions, either inside or outside the ICU.

## DISCUSSION

The findings of our study indicated that frailty of the elderly patients had no impact on the nursing workload, given that the NAS scores of frail elderly people did not differ from the scores of non-frail elderly patients, either by comparing the overall score or the score determined in each NAS category/item. However, it is important to emphasize that, regardless of the frailty condition, both groups required a high nursing workload on the first day in the ICU. In the daily practice of ICUs, in general, newly admitted patients receive several invasive therapeutic procedures and complex care; also, more attention to the family is necessary for the most critical moments, factors that demand a high nursing workload. The perception that frailty in the elderly helps increase the severity of illness may have resulted in higher SAPS 3 scores in frail elderly patients. However, a positive correlation between frailty, illness severity, and consequent increase in nursing workload, empirically observed in ICU clinical practice, was not observed in our study.

Considering that few studies are available about frailty among the elderly and the impact of this condition on the demand for nursing care in ICUs, mainly regarding the nursing workload, the comparison of results became a challenge.

Studies that have analyzed the nursing workload related to elderly care in the ICU, as measured by the NAS, have presented high workloads^(8,9,21)^. However, to study the association of the condition of frailty, illness severity, and nursing workload in a context of high prevalence of elderly people in ICUs and predominance of frailty, sample size calculation and multicenter strategies are important factors.

Frailty is a common condition among elderly people, with 20% prevalence at 65 years of age. It is associated with adverse responses to diseases, leading to higher morbidity and hospitalization rate and demanding different levels of care complexity^(12,22)^. In our study, a high prevalence of frail elderly people in the ICU was observed.

In the studied sample, some aspects of the NAS items that were more frequently observed in the group of frail elderly people characterized a clinical condition that required nursing care and, consequently, generated nursing workload. One of these aspects was the higher frequency of both non-invasive and invasive mechanical pulmonary ventilation and tracheostomy in frail patients than in non-frail ones. It is known that the state of frailty is related to physiological changes such as sarcopenia, immunological changes, and inflammatory processes^(23,24)^. In the analysis of frailty, the impairment of the physical domain, with an increase in the severity of the disease, may cause the need for these and other supporting therapies to maintain their clinical conditions. The presence of frailty among patients on mechanical ventilation is associated with increased hospital mortality rate, extubation failure, and the need for tracheostomy^(25)^. The need for more frequent non-invasive ventilation in frail elderly people was observed in a sample of critically ill patients analyzed in a Spanish study^(26)^. However, results of systematic reviews did not show statistically significant differences regarding the use of mechanical ventilation or vasopressors^(4,27)^ between frail and non-frail elderly people. Indirectly, this aspect was observed in our study through the NAS in the ventilatory and cardiovascular support items, in which the proportion of elderly people was similar regardless of the frailty condition.

This study allowed the analysis of the illness severity as well as other nursing activities involved in the NAS. Regarding administrative tasks and family support, our study showed a higher frequency of scores for frail elderly patients. This aspect may be related to the fact that frail elderly people present deeper physical impairment, and social and psychological problems when compared to non-frail elderly people. Feelings of sadness and depression were observed among frail elderly people in an Australian ICU using the Edmonton Frailty Scale and the Clinical Frailty Scale^(28)^. In the systematic review that analyzed the relationship between frailty in the elderly and depression, the presence of comorbidity and depressive symptoms increase the risks for frailty^(29)^. In addition to psychological aspects, social factors also have a direct impact on nursing care since the lack of a support network for the elderly can cause suffering during hospitalization. A study conducted in the city of São Paulo found a higher proportion of frail elderly people without a partner^(30)^. Thus, frail elderly people may feel sadder, more nervous or anxious, as they miss having people by their side. This condition may increase the nursing workload.

In our study, the NAS score on the first day of hospitalization in the ICU was adopted to analyze the nursing workload, representing the moment of higher instability due to the illness severity, which in frail elderly people can be higher due to physical impairment. However, in view of the findings, it is important to consider that the critical period, corresponding to the first week in this study, was not sufficient to measure the demand for nursing care considering the frailty condition. Perhaps the NAS application during the ICU period may show that elderly people with a chronic critical illness demand a higher nursing workload when compared to the critical or acute period. Also, the analysis of NAS scores in their categories/items, rather than a proportion, should be considered.

This study has limitations related to the absence of sample size calculation based on the prevalence of frail elderly people in the ICU and data collection in a single ICU. The use of an informer to answer the TFI questions for frailty assessment may have been another limiting factor, since the scale is self-reported. Thus, under- or overestimated responses from informers could have affected the accuracy of frailty assessment and contributed to refusal to participate in the study, especially among more severe patients.

## CONCLUSION

In conclusion, frailty among elderly people did not increase the nursing workload in the ICU. Regardless of the frailty condition, the nursing workload in elderly care was high on the first day in the ICU.

For future studies, we suggest the application of multidimensional scales to assess frailty immediately upon admission of the elderly patient to the hospital, prior to sedation and intubation, i.e., while the patient has conditions to answer the questions, so that there is no need to include an informer. Another aspect to be considered is related to the NAS, observing, in addition to the score, the number of nursing professionals required for elderly care.

## References

[1] Sieber CC (2017). Frailty: from concept to clinical practice. Exp Gerontol..

[2] De Biasio JC, Mittel AM, Mueller AL, Ferrante LE, Kim DH, Shaefi S (2020). Frailty in critical care medicine: a review. Anesth Analg..

[3] Fried L, Tangen CM, Walston J, Newman AB, Hirsch C, Gottdiener J (2001). Cardiovascular Health Study Collaborative Research Group. Frailty in older adults: evidence for a phenotype. J Gerontol A Biol Sci Med Sci..

[4] Muscedere J, Waters B, Varambally A, Bagshaw SM, Boyd JG, Maslove D (2017). The impact of frailty on intensive care unit outcomes: a systematic review and meta-analysis. Intensive Care Med..

[5] Alghamdi MG (2016). Nursing workload: a concept analysis. J Nurs Manag..

[6] Queijo AF, Padilha KG (2009). Nursing Activities Score (NAS): adaptação transcultural e validação para a língua portuguesa. Rev Esc Enferm USP..

[7] Miranda DR, Nap R, Rijk A, Schaufeli W, Iapichino G. (2003). Nursing Activities Score (NAS). Crit Care Med..

[8] Sousa CR, Gonçalves LA, Toffoleto MC, Leão K, Padilha KG (2008). Preditores da demanda de trabalho de enfermagem para idosos internados em unidade de terapia intensiva. Rev Lat Am Enfermagem..

[9] Altafin JA, Grion CM, Tanita MT, Festti J, Cardoso LT, Veiga CF (2014). Nursing Activities Score e carga de trabalho em unidade de terapia intensiva de hospital universitário. Rev Bras Ter Intensiva..

[10] Simões JL, Sa-Couto P, Simões CJ, Oliveira C, Santos NM, Mateus J (2021). Nursing workload assessment in an intensive care unit: a 5-year retrospective analysis. J Clin Nurs..

[11] Kim SW, Yoon SJ, Choi JY, Kang MG, Cho Y, Oh IY (2017). Clinical implication of frailty assessment in older patients with atrial fibrilation. Arch Gerontol Geriatr..

[12] Dent E, Kowal P, Hoogendijk EO (2016). Frailty measurement in research in research and clinical practice: a review. Eur J Intern Med..

[13] Gonçalves LA, Garcia PC, Toffoleto MC, Telles SCR, Padrilha KG (2006). The need for nursing care in intensive Care Units: daily patient assessment according to the Nursing Activities Score (NAS). Rev Bras Enferm..

[14] Santiago LM, Luz LL, Mattos IN, Gabbens RJJ (2012). Cross-cultural adaptation of the Tilburg Frailty Indicator (TFI) for use in the Brazilian population. Cad Saude Publica..

[15] Charlson ME, Pompei P, Ales KL, MacKenzie CR (1987). A new method of classifying prognostic comorbidity in longitudinal studies: development and validation. J Chronic Dis..

[16] Moreno RP, Metnitz PGH, Almeida E, Jordan B, Bauer P, Campos RA (2005). SAPS 3 Investigators. From evaluation of the patient to evaluation of the intensive care unit. Part 2: development of a prognostic model for hospital mortality at ICU admission. Intensive Care Med..

[17] Stricker KH, Kimberger O, Brunner L, Rothen HU (2011). Patient satisfaction with care in the intensive care unit: can we rely on proxies?. Acta Anaesthesiol Scand..

[18] Conishi RMY, Gaidzinski RR (2007). Nursing Activities Score (NAS) como instrumento para medir carga de trabalho de enfermagem em UTI adulto. Rev Esc Enferm USP..

[19] Brasil, Ministério da Saúde, Agência Nacional de Saúde Suplementar – ANS (2013). Média de permanência UTI adulto.

[20] Castro MCN, Dell’Acqua MCQ, Unger IC, Cyrino CMS, Almeida PMV (2018). Gravidade e carga de trabalho de enfermagem em pacientes candidatos à vaga na UTI. Esc Anna Nery..

[21] Ferretti-Rebustini REL, Nogueira LS, Silva RCG, Poveda VB, Machado SP, Oliveira EM (2017). Aging as a predictor of nursing workload in intensive care unit: results from a Brazilian Sample. Rev Esc Enferm USP..

[22] Pijpers E, Ferreira I, Stehouwer CD, Nieuwenhuijzen Kruseman AC (2012). The frailty dilema: review of the predictive accuracy of mayor frailty scores. Eur J Intern Med..

[23] Sloane PD, Cesari M (2018). Research o Frailty: continued progress, continued challenges. J Am Med Dir Assoc..

[24] Robertson DA, Savva GM, Kenny RA (2013). Frailty and cognitive impairment: a review of the evidence and causal mechanisms. Ageing Res Rev..

[25] Fernando SM, McIsaac DI, Rochwerg B, Bagshaw SM, Muscedere J, Munshi L (2019). Frailty and invasive mechanical ventilation: association with outcomes, extubation failure, and tracheostomy. Intensive Care Med..

[26] López Cuenca S, Oteiza López L, Lázaro Martín N, Irazabal Jaimes MM, Ibarz Villamayor M, Artigas A (2019). Frailty in patients over 65 years of age admitted to Intensive Care Units (FRAIL-ICU). Med Intensiva..

[27] Xia F, Zhang J, Meng S, Qiu H, Guo F (2021). Association of frailty with the risk of mortality and resource utilization in elderly patients in intensive care units: a meta-analysis. Front Med..

[28] Darvall JN, Greentree K, Braat MS, Story DA, Lim WK (2019). Contributors to frailty in critical illness: multi-dimensional analysis of the clinical frailty scale. J Crit Care..

[29] Vaughan L, Goveas J, Corbin A (2015). Depression and frailty in later life: a systematic review. Clin Interv Aging..

[30] Duarte YAO, Nunes DP, Andrade FB, Corona LP, Brito Tábatta RP, Santos JLF (2018). Frailty in older adults in the city of São Paulo: prevalence and associated factors. Rev Bras Epidemiol..

